# An Ancient Divide in a Contiguous Rainforest: Endemic Earthworms in the Australian Wet Tropics

**DOI:** 10.1371/journal.pone.0136943

**Published:** 2015-09-14

**Authors:** Corrie S. Moreau, Andrew F. Hugall, Keith R. McDonald, Barrie G. M. Jamieson, Craig Moritz

**Affiliations:** 1 Field Museum of Natural History, Department of Science and Education, 1400 South Lake Shore Drive, Chicago, Illinois 60605, United States of America; 2 Museum Victoria, GPO Box 666, Melbourne, VIC 3001, Australia; 3 Queensland Parks and Wildlife Service, Northern Region, Atherton, QLD 4883, Australia; 4 University of Queensland, School of Biological Sciences, Brisbane, QLD 4072, Australia; 5 Australian National University, Research School of Biology & Center for Biodiversity Analysis, Canberra, ACT 2601, Australia; Onderstepoort Veterinary Institute, SOUTH AFRICA

## Abstract

Understanding the factors that shape current species diversity is a fundamental aim of ecology and evolutionary biology. The Australian Wet Tropics (AWT) are a system in which much is known about how the rainforests and the rainforest-dependent organisms reacted to late Pleistocene climate changes, but less is known about how events deeper in time shaped speciation and extinction in this highly endemic biota. We estimate the phylogeny of a species-rich endemic genus of earthworms (*Terrisswalkerius*) from the region. Using DEC and DIVA historical biogeography methods we find a strong signal of vicariance among known biogeographical sub-regions across the whole phylogeny, congruent with the phylogeography of less diverse vertebrate groups. Absolute dating estimates, in conjunction with relative ages of major biogeographic disjunctions across Australia, indicate that diversification in *Terrisswalkerius* dates back before the mid-Miocene shift towards aridification, into the Paleogene era of isolation of mesothermal Gondwanan Australia. For the Queensland endemic *Terrisswalkerius* earthworms, the AWT have acted as both a museum of biological diversity and as the setting for continuing geographically structured diversification. These results suggest that past events affecting organismal diversification can be concordant across phylogeographic to phylogenetic levels and emphasize the value of multi-scale analysis, from intra- to interspecies, for understanding the broad-scale processes that have shaped geographic diversity.

## Introduction

Geologic and climatic oscillation events are likely to have profound effects on both rates of speciation and current distributions of taxa. The processes and timelines involved have long intrigued biologists [1] [2] [3] [4]. Moreover, there has been a long-standing attempt to explain and understand the high species richness of the tropical rainforests [5] [6] [7] [8] and whether the rainforests act as a museum or cradle for biological diversity [9] [10] [11] [12]. More generally, there is growing appreciation of how long-term processes of landscape change and phyletic evolution shape regional species pools and geographically structured diversity [11] [13]. Explanations for the high biological diversity of the tropics and the current distributions of species have included biotic and abiotic factors with no single variable accounting for the patterns we observe. One suite of related factors that have been proposed to explain some of the observed patterns are the effects of Pleistocene or older climate change in the tropics, the resulting refugia, and the impacts of these events on speciation [14] [15], though such refuge-based models have proved controversial [16] [17]. In this paper we explore whether climate-driven vicariance, as measured by biogeographic structuring (ancestral ranges) of sister clades within the phylogeny, has shaped long-term speciation in a system with a well-characterized history of late Quaternary contraction and refugia: The rainforests of the Australian Wet Tropics.

The Australian Wet Tropics (AWT) are the largest surviving remnant of Gondwanan rainforest, which once dominated the continent of Australia [18]. Climatic modeling and paleoecological data [19] [20] [21] [22] [23] and previous research on montane taxa [24] [25] [26] have shown that the currently near continuous rainforest was sundered into disconnected refugia as a result of late Quaternary climatic processes. These late Quaternary processes, especially in the mid- late Pleistocene [20] [27], had profound effects on current patterns of species richness and phylogeographic diversity [21] [25] [26] [28]. Based on the growing body of research from this region, there is support for the hypothesis that these refugia are the result of late Quaternary climate changes [24] [29] [30], perhaps undergoing extreme contraction, and for many of the groups that are found in these regions, large-scale extinction. Not only have phylogeographic studies provided evidence of Pleistocene climatic events leaving discrete genetic signatures on modern populations, there is mounting evidence that the major “breaks” in genetic diversity (including across the Black Mountain Corridor) typically predate the Last Glacial Maximum ([31]: late-Miocene or early Pliocene; [32]: Pliocene; [33]: pre-Pleistocene; [28]: Late Pliocene or Early Pleistocene; [34]: pre-Pleistocene; [26]: Pliocene 2.3–4.6 Ma). Interestingly, historic biogeographic features within the AWT may not have the same impact on related taxa, as was found for two species of closely related rainforest trees with overlapping habitats and differing signature of the impact of the geographic features and genetic diversity [34]. These previous studies highlight variation among species in the spatial and temporal scale of response.

The habitat contractions of the AWT are thought to be due to the long-term aridification of the Australian continent, which began during the mid-Miocene, through the Pliocene and continued with the high amplitude oscillation of the mid-late Pleistocene [18] [35]. The resulting isolation and local reduction of rainforest tracts doubtlessly had major effects on the biological diversity of this region. The contractions of the forests would clearly have cascading effects for taxa that had close ecological and evolutionary relationships with the rainforest themselves [36] [37] [38]. The escarpments and montane areas that harbor the endemic fauna of the Australian Wet Tropics are, in general, an ancient land formation. In this context, it is possible that rainforest contractions much deeper in time (e.g. mid-Miocene) have shaped current patterns of diversity. If true, how does this affect deeper phylogenetic splits? If climate driven contractions of rainforest over Plio-Pleistocene (or even mid- late Miocene or older) affected speciation processes, then phylogenies of species-rich endemic genera should show geographic structure at these deeper evolutionary scales analogous to phylogeographic studies observed within species. For most vertebrate genera unraveling the geography of speciation associated with longer time scales has not been possible because few, closely related clades of species are found within the AWT and most genera have low species diversity [39]. A prominent exception is microhylid frogs in the genus *Cophixalus*, for which spatial phylogenetic pattern is congruent with geography [40].

**Fig 1:**
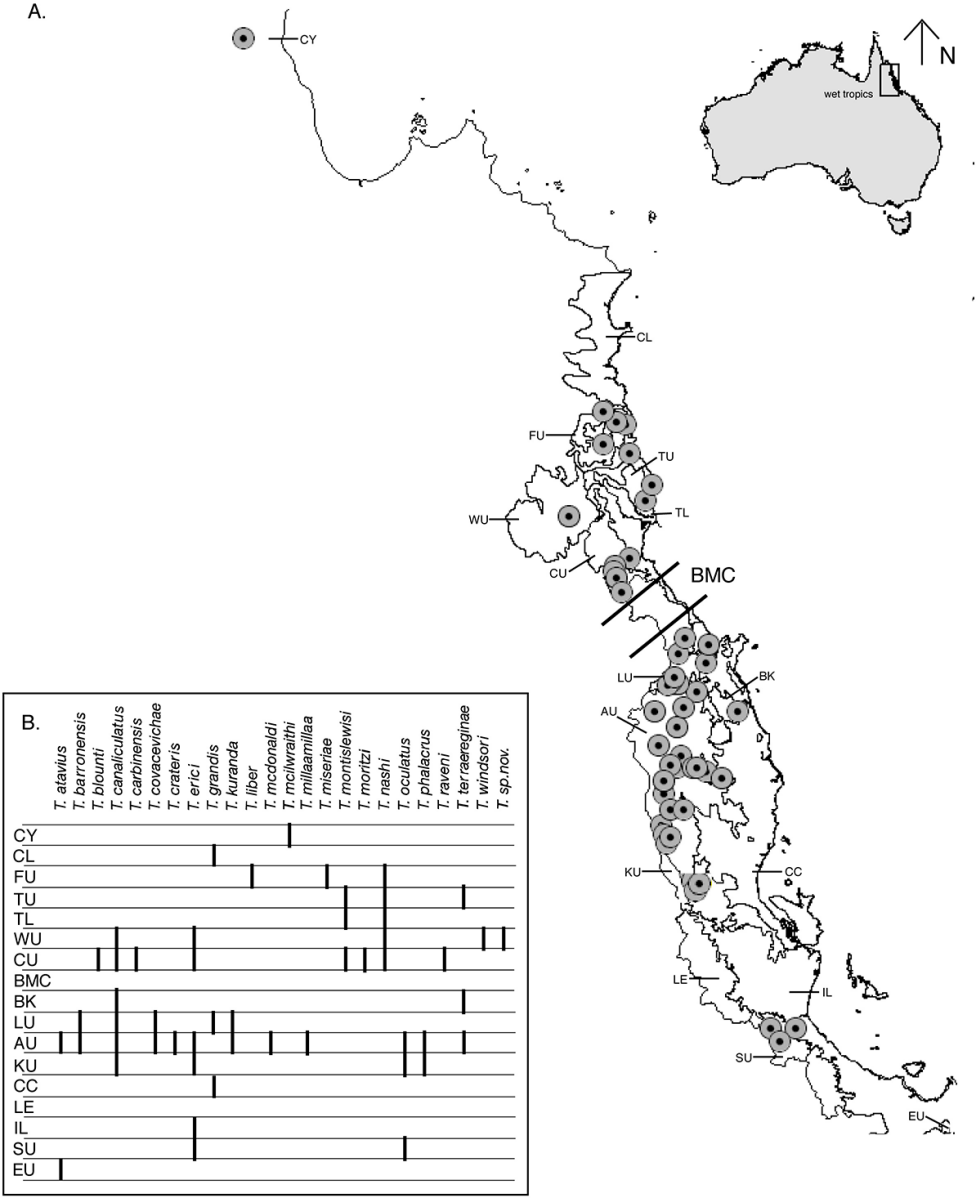
Distribution data for species of *Terrisswalkerius* earthworms within the Australian Wet Tropics with biogeographic subregions after Williams *et al*. [82]. The AWT are found between latitudes 15°39' south and 19°17' south, and longitudes 144°58' east and 146°27' east. **A.** Map showing sample collection locations for species of *Terrisswalkerius*. **B.** Species presence within each subregion for all known collections of *Terrisswalkerius*.

Earthworms have been largely overlooked in studies of historical biogeography and phylogeography and have rarely been subjected to molecular analysis until recently [41] [42] [43] [44] [45] [46] [47] [48] [49] [50] [51] [52] [53]. As a diverse, Australian Wet Tropics endemic and rainforest-restricted group, earthworms of the genus *Terrisswalkerius* provide a promising system with which to address questions of long-term historical processes in the AWT (Fig 1). Like terrestrial snails that have been highly informative for phylogeography [25] [54], the earthworms are expected to have limited dispersal ability and to have been less prone to late Pleistocene local extinctions because of their large overall distributions and diversity. As inhabitants of moist soils and rotten logs with presumed environmental buffering, their biogeographic history may provide a rich view of the long-term historical processes that have shaped the region. *Terrisswalkerius* earthworms are striking due to their size (ranging from 24mm- 317mm) and often bright colors. The 25 species of *Terrisswalkerius* are distributed in the wet tropical rainforests of eastern North Queensland, from the Paluma Range, near Townsville north to the isolated rainforest of the McIlwraith Range in the Cape York Peninsula. Jamieson [42] reviewed the biology, distribution, and morphology of all known species of *Terrisswalkerius* and wide-scale molecular phylogenetic analyses [44] [53] confirmed that the genus is a member of the annelid family Megascolecidae. An unpublished new species is included in the present study and a further species, *T*. *leichhardti*, was described by Jamieson *et al*. [55].

As these endemic earthworm species are intimately associated with forest environments, they may be expected to track this long continuous history of decimation of the Gondwanan mesothermal rainforests that once dominated Australia, of which the Wet Tropics in considered to be the largest surviving remnant. Thus broadly, the current endemic species distributions of *Terrisswalkerius* may reflect a balance of 1) re-structuring of old, geographically dispersed diversity into persistent and much reduced regions; and 2) biogeographic speciation among regions. This balance might be weighed by assessing (by ancestral range reconstruction) the signal of vicariant speciation consistent with Australian Wet Tropics biogeography, compared to more complex idiosyncratic patterns expected from restructuring of ancestral diversity.

Here we estimate the phylogenetic structure of the AWT endemic earthworm lineage, *Terrisswalkerius*, overlaying this with current species distributions and ancestral range reconstruction to infer the effect of known barriers deduced from previous phylogeographic studies on long-term speciation processes. We also provide a provisional estimate for the timing of the diversification of the genus and the results are compared to recent phylogenetic analyses of other endemic but lower diversity taxa.

## Materials and Methods

We amplified and sequenced DNA fragments from 16 species of *Terrisswalkerius* (34 specimens) and an additional 34 megascolecid outgroup taxa (68 specimens total). The nine species of *Terrisswalkerius* that were not included in this study are known only from the type collections and were not preserved in a manner likely to yield useful DNA sequences, excepting *T*. *oculatus*, although known from three localities, was not obtainable for sequencing. All collections were made under the auspices of the Department of the Environment (Queensland, Australia) in which one of the authors (KRM) was an officer and no additional permissions were required. The species studied are not on any threatened or endangered lists but we hope that this paper will encourage their protection. All worms were humanely euthanized in dilute ethanol following all protocols of the Department of the Environment (Queensland, Australia). All included specimens were sequenced for the mitochondrial (mt) 12S rRNA gene, with nested coverage for the following additional genes: mt 16S rRNA, mt DNA *COII*, and nuclear 28S rRNA [see S1 File for sequencing details and S1 Table for GenBank accession numbers].

### Phylogenetic inference

The final concatenated data matrix comprised 695 sites of nuclear 28SrDNA, 315 and 435 sites of mitochondrial 12S and 16S rDNA respectively, and 558 sites of mitochondrial *COII*. Phylogenetic analyses were conducted on this data matrix using maximum parsimony, and maximum likelihood and Bayesian inference using partitioned GTR+Γ+I models for each gene region (Modeltest 3.06: [56]). Parsimony searches were performed with PAUP*4.0b10 [57] using the random stepwise addition option of the heuristic search for 500 replicates with tree bisection-reconnection (TBR) branch swapping, collapse of zero-length branches, and equal weighting of all characters, with bootstrap analyses (bs) using the closest stepwise addition of the heuristic search for 500 replicates. Maximum likelihood phylogenetic inference was conducted with RAxML v7.2.6 [58] using 200 fast bootstraps. Bayesian analyses were performed with MrBayes v3.1.1 [59], using four 20 million generation Markov chains, sampled every 100 generations after a burn-in of 100,000 generations for Bayesian posterior probability (bpp) estimation. Convergence of chains was confirmed in all Bayesian analyses by examination of the average standard deviation of split frequencies. Additional analyses were also conducted with BEAST v1.4.8, as describe below, and GARLI v0.951 [60] (see SI for details).

To test alternative hypotheses for the monophyly of *Terrisswalkerius* a constraint tree search was implemented and the Shimodaira-Hasegawa test [61] was executed to investigate significant differences in tree lengths. This test was performed using RELL with 10,000 bootstrap replicates, and the results evaluated as a one-tailed test.

### Biogeographic reconstruction

We employed biogeographic history DEC-LAGRANGE [62] [63] and S-DIVA [64] methods to infer the ancestral ranges through the phylogeny of *Terrisswalkerius*. In addition to individual node ancestral state reconstruction we summarized the relative amount of vicariance across the whole phylogeny. In LAGRANGE (version 20110117) this was done by summing the relative probabilities of ancestral node solutions that split into two mutually exclusive regions. For example, if the ancestral range is ABC and the daughter lineages ranges are AB and C. This is similar to the approach of Beaulieu *et al*. [65]. In S-DIVA nodes were scored as vicariance according to the most likely reconstruction. For the purposes of these summary biogeographic analyses we focused on a sub-tree of 23 nodes representing all the species and major lineages. Eight biogeographic regions were defined. Tests of maximum range limits 2–3 and dispersal constraints had only minor effects on the overall result (cf. [66]) and we use the results for the analysis presented in full in SI.

### Divergence estimation

We performed a relaxed-clock dating using the combined data with a normal rate prior on the *COII* partition and two major calibration constraints. The analysis was run in BEAST v1.4.8 [67] with uncorrelated lognormal clock model, Yule speciation prior, and partitioned GTR+Γ+I models for each gene region. The analysis was calibrated using a truncated normal rate prior on the *COII* partition (min = 0.005; mean = 0.01; SD = 0.01 per lineage), and a mimimum 50 million years ago (Ma) divergence for the Acanthodrilinae and 80 Ma for the Megascolecidae. These represent a summary of *COII* rates used for a range of invertebrates including oligochaetes [50] [68] [69], and major Earth biogeography limits for these widely distributed oligochaete groups [44] [70]. Two 20,000,000 step chains were run with 1/1000 sampling and 10% burn-in, leaving 18,000 posterior samples. All parameter ESS were greater than 200. Our results are comparable to previous oligochaete dating using similar data: Novo *et al*. [50], who estimated much the same age of ~70 Ma for the Lumbricidae (equivalent to our *Criodrilus-Lumbricus* split); and relative ages of Acanthodrilinae-Megascolecinae taxa by Buckley *et al*. [49]. The full relaxed-clock tree with calibration points and outgroups is shown in S1 Fig.

In addition to considering the absolute ages returned from the relaxed-clock dating, we also considered ages relative to several major disjunctions pertinent to deep Austral biogeography: the WT-Cape York *Terrisswalkerius mcilwraithi* split, the WT–SE Queensland (Qld) split in the sister group *Fletcherodrilus*, the SE Qld–Tasmania divergence in the *Digaster-Diporochaeta*, and the apparent absence of the group from New Guinea. These relate to the Miocene timescale of the major environmental shift to fragmentation and attenuation of mesic mesothermal environments [19] [20] [71], and age of New Guinean landmasses [72].

## Results

**Fig 2:**
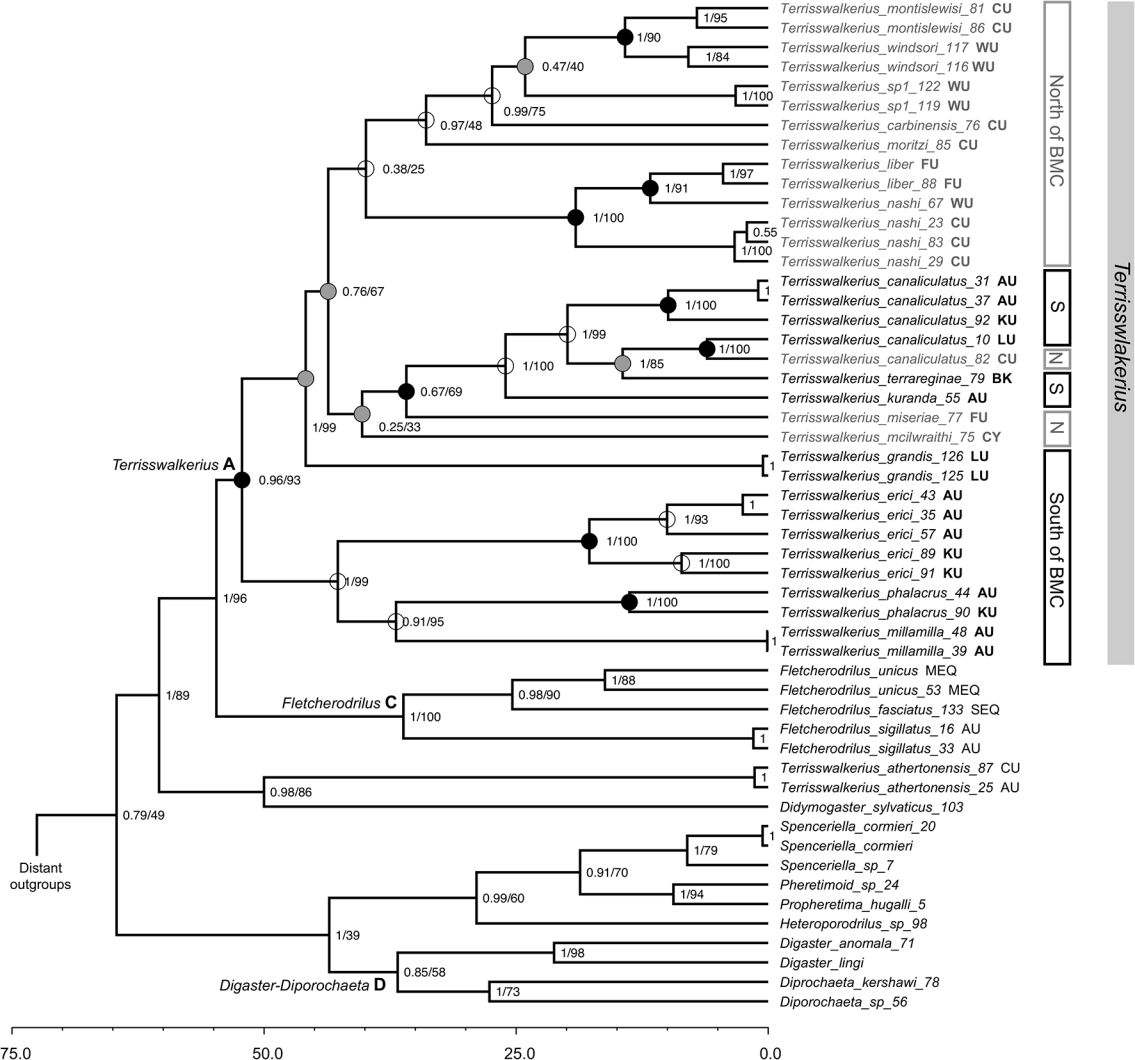
BEAST relaxed-clock maximum clade credibility phylogeny, showing BEAST posterior probabilities followed by RAxML bootstrap supports (PP/BS as percent; some terminal nodes show only PP to avoid clutter) based on the combined data matrix. *Terrisswalkerius* taxa north of the Black Mountain Corridor (BMC) in grey. Node circles indicate biogeographic analyses: grey and black filled circles indicate inferred ‘vicariance’ by respectively either or both DEC and DIVA methods. Key dating nodes indicated. Divergence scale in Ma. See Jamieson *et al*. [44] and Buckley *et al*. [49] for more details on higher classification. Full tree with distant outgroups shown in S1 Fig.

All phylogenetic methods recovered similar topologies for the relationships within *Terrisswalkerius* and we present the BEAST consensus tree with ML and Bayesian support values in Fig 2 (see S1 Fig for the full tree with all distant outgroup taxa included). The major framework of the tree (Fig 2) is generally congruent between mitochondrial and nuclear gene data (see, however, [44], where 28S rDNA showed poor resolution compared with the combined nuclear and mitochondrial analysis; S3 and S4 Figs). *Terrisswalkerius* is recovered as monophyletic with the exception of *T*. *athertonensis*, the inclusion of this species being rejected by the SH test (delta lnL = 36.4, p = 0.0057). The position of *Didymogaster* Fletcher 1886 is somewhat equivocal with mtDNA being responsible for placement within *Terrisswalkerius sensu lato*, where *T*. *athertonensis* is included. All data unequivocally support *Fletcherodrilus* Michlaelsen 1891 as the sister group of *Terrisswalkerius*, if *T*. *athertonensis* is excluded, as also shown in the maximum likelihood analysis of Jamieson *et al*. [44]. The present and previously published results [42] [44] suggest that *T*. *athertonensis* should be placed in a separate, cryptic genus. In a morphological cladistic analysis [73] it grouped with *T*. *oculatus*, embedded among *Terrisswalkerius* species but the morphological tree shows little agreement with molecular analyses. *Terrisswalkerius* Jamieson 1994 remains current pending further resolution and confirmation of the separate generic position of *T*. *athertonensis*. *Terrisswalkerius s*.*s*. is mostly well resolved with the exception of basal relationships involving *T*. *macilwraithi* and the *T*. *nashi-liber* lineage.

Although, in general, morphologically defined species are recovered as monophyletic, we do recover two paraphyletic species (*T*. *canaliculatus* and *T*. *nashi*), which may suggest that further taxonomic revision is needed in this diverse and understudied group. However, as implied by Jamieson [42], *T*. *nashi* would be monophyletic if *T*. *liber*, differing chiefly in approximation of genital pores, is included within *T*. *nashi*.

### Biogeographic reconstruction

In examining the biogeographic structure of the *Terrisswalkerius* phylogeny, we find a clade containing three species (*T*. *erici*, *T*. *millamilla*, and *T*. *phalacrus*) found in the Atherton Uplands (AU) and Kirrama Uplands (KU), both south of the Black Mountain Corridor (although for some taxa the BMC split extends south of LU (Lamb Uplands); see [74], as sister to the remaining taxa, comprising both northern and southern taxa (Fig 2).

Summarized across 23 nodes representing all the species and major lineages, DEC-LAGRANGE recorded 9 nodes as vicariance and S-DIVA 14 nodes (Fig 2; S2 Fig; S2 Table). For *Terrisswalkerius s*.*s*. (node A) the DEC marginal likelihood for vicariance was 0.53, which was also the most likely solution in S-DIVA. Overall, vicariance accounts for nearly half of the nodes, and not only among obvious sister tips but also through the depth of the phylogeny. This suggests that the regional biogeography of the Australian Wet Tropics has been an important component in diversification over an extended period of time.

Both DEC and DIVA reconstruct the most likely solution for node A (the ancestor of *Terrisswalkerius*) as a split between AU and either LU or CU (S2 Table). The extant species are then divided into two distinct clades with one clade found exclusively south of the BMC and the other with a wide distribution including all of the taxa north of the BMC and the extra-limital Cape York *T*. *macillwraithi*. Within this ‘northern’ clade the history is complex but with considerable signal of large-scale ‘north-south’ vicariance. Considering the biogeographic analyses and the known current distributions for species, we find what may have been expansions back across the BMC (although resolution is weak in this part of the topology) and this is probably the case for *T*. *canaliculatus*, *T*. *erici*, *T*. *grandis*, and *T*. *terraereginae*. We also find clear repeated diversification within individual regions of the AWT such as in the Atherton Uplands (AU; 13 species), Carbine Uplands (CU; eight species), Kirrama Uplands (KU; four species), Lamb Uplands (LU; six species), and Windsor Uplands (WU; five species). The overall pattern we recover in the AWT endemic *Terrisswalkerius* earthworms appears to be a combination of deep ‘north-south’ splits and more recent sub-regional diversification.

### Absolute and relative divergence dating

**Fig 3:**
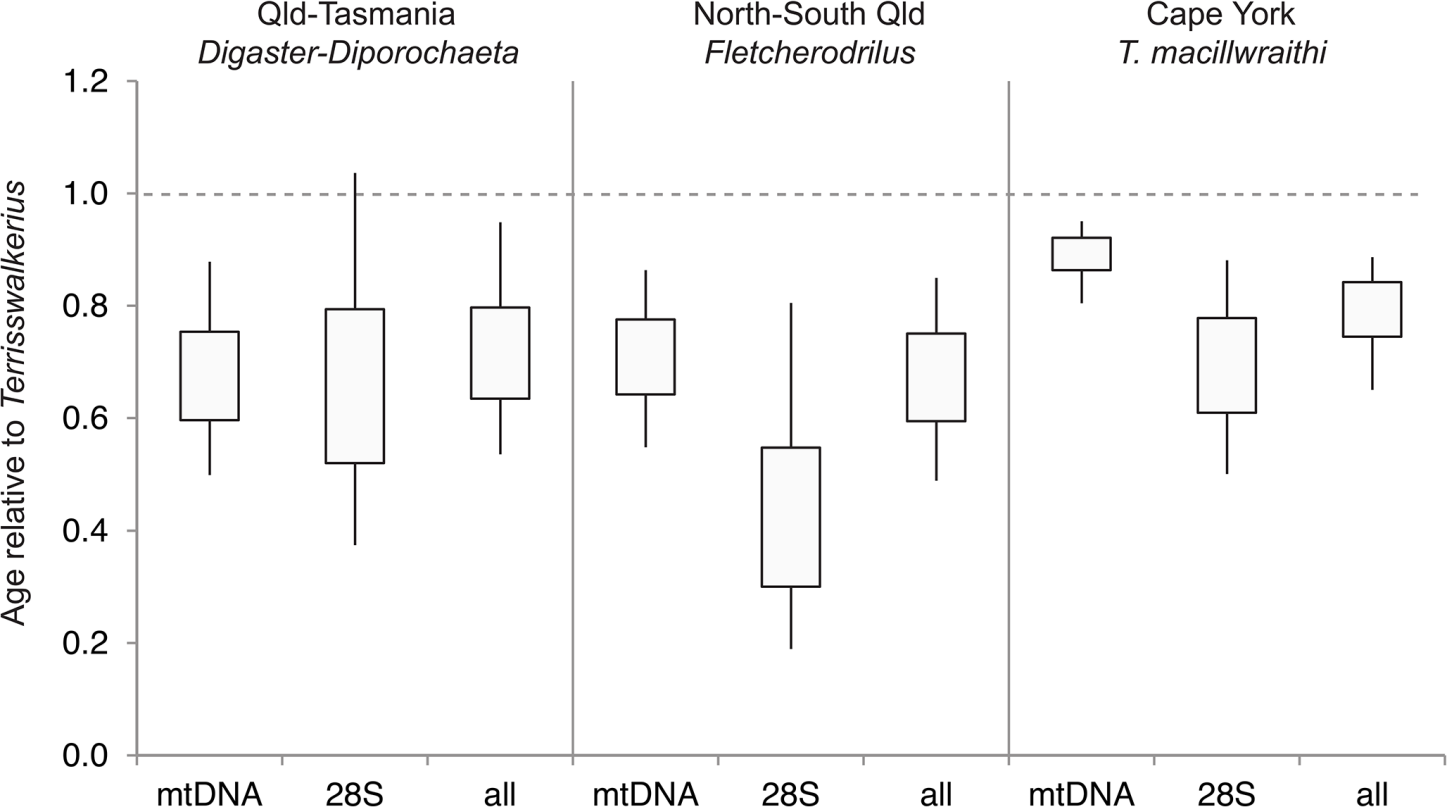
Ages of various groups relative to monophyletic *Terriwalkerius* (node A). Quartile and 95% CI BEAST posterior distributions. *Digaster-Diporochaeta* (node D) represents taxa spread from SE Queensland to Tasmania; *Fletcherodrilus* (node C) represents taxa spread across the Burdekin and St. Lawrence Gaps from the AWT to SE Qld; Cape York *T*. *macillwraithi* represents the Laura Gap. Estimates use three different data matrices: mtDNA only, 28SrDNA only, and combined (all) (see SI for further details).

Relaxed-clock dating estimated the age of monophyletic members of *Terrisswalkerius* (labeled “A” in Fig 2; S1 Fig) to be 52 Ma (with 95% CI of 31–84 Ma), the separation of the Cape York species, *T*. *mcilwraithi* at 40 Ma (21–58 Ma), the North-South Queensland split in *Fletcherodrilus* at 36 Ma (12–46) and the Qld-Tasmania split in the *Diporochaeta* group at 36 Ma (19–62 Ma). There is considerable uncertainty in these estimates due to limited calibration information and high level of molecular change in the mitochondrial genes. However, relative dating should be less sensitive to these limitations [49] [75]. Significantly, the Cape York *T*. *mcilwraithi*, North-South Queensland *Fletcherodrilus* and the Qld-Tasmania *Diporochaeta* splits are all younger than *Terrisswalkerius* s.s. (node A), and this pattern is consistent considering mitochondrial or nuclear data alone (Fig 3). It is also worth noting that the clade is around five times older than typical intra-specific phylogeography (Fig 2).

This inference places extant monophyletic *Terrisswalkerius* well into the Paleogene era of isolation of mesothermal Gondwanian Australia [19] [71] [76], and even the lower confidence interval (31 Ma) places a substantial proportion of the Australian Wet Tropics endemic diversity pre-dating the mid-Miocene major shift towards fragmentation and aridification [20] [71]. This can be further put in context by considering the relative age of phylogenetic splits spanning deep Austral biogeography (Fig 2). While each of these splits may not directly relate to historically driven eco-geographic divergence processes, they set a maximum (e.g. an overestimation due to extinction or under-sampling) and therefore emphasize the age of the AWT endemic *Terrisswalkerius* diversity as likely pre-dating the mid-Miocene major shift towards fragmentation and aridification. This can also explain their apparent absence from any New Guinean landmass, which (at ca. 10–15 Ma; [72]) likely post-dates these major historical eco-geographic divisions.

## Discussion

Geologic and climatic oscillation events are expected to have profound effects including attenuation and extinction on less mobile organisms; the genetic signatures of these events are apparent in the endemic Australian Wet Tropics earthworms. The substantial component of geographical speciation between regions–vicariance–estimated across the phylogeny as a whole and fairly apparent in sister tip lineage phylogeography (Fig 2), indicates that the biogeographical structure of the AWT rainforests has played a significant role in diversification of *Terrisswalkerius*.

In the case of these earthworms, morphological conservatism hides the deep evolutionary history of the lineage, as there are few morphological characters to distinguish these species, which lack the genital markings that show high specificity in other megascolecid genera. Although we infer a middle Paleogene age (ca. 52 Ma) for the genus *Terrisswalkerius*, dating divergence times for lineages can be challenging in the absence of fossil or biogeographic information, but clearly molecular divergences are high. Relatively, *Terriwalkerius* is five times older than typical intra-specific phylogeography (Fig 2) and older than major biogeographic divisions (Fig 2; S2 Fig; S3 Table), consistent with it being of at least Olio-Miocene age.

Our finding of the signature of an ancient north-south division in *Terrisswalkerius* suggests the ‘Black Mountain Corridor’ may be a much older biogeographic barrier than had been previously proposed and played a role in AWT diversification prior to the Pleistocene-era phylogeography of previous vertebrate studies. There is now mounting evidence from phylogenetic analyses that the BMC barrier predates the Last Glacial Maximum [32] [33] [74] and our findings significantly push back the date for this barrier and add to this growing body of work. The distantly isolated Cape York species, the complex biogeographic history of the ‘northern’ lineage, and the sheer age of the whole group, all suggest a complex history coinciding with the drying of the Australian continent, which is thought to have begun with the major ice-sheet expansion of the late-Paleogene [76] and continued from the mid-Miocene [18]. Thus for *Terrisswalkerius*, *in situ* AWT endemic processes continued all the while the greater mesic mesothermal Gondwanian world shrank and fragmented around it, leaving the isolated ‘island world' of the Australian Wet Tropics.

Although the AWT are a very well studied region, the area to the north is less explored and the effect of long-term aridification and Pleistocene cycles on the isolated rainforest patches of the Cape York peninsular region are not well known. In addition the biological diversity of the Cape York (CY) area is understudied, but available evidence suggests that this region contains phylogenetic relics *i*.*e*. deep branch taxa [39]; this is the case in *Cophixalus* microhylid frogs [40], leaf-tailed geckos [77], camaenid snails [54], dung beetles [78] and now within the *Terrisswalkerius* earthworms (*T*. *mcilwraithi*: Fig 2). Our data recover the northern Cape York species, *T*. *mcilwraithi*, as an early lineage separating at ~ 26 Ma, within the primarily northern clade which is suggestive of long-term isolation and/or extinction of species within this clade. The hiatus in occurrence of *Terrisswalkerius* between CL and CY appears to be real as extensive collecting by KRM in the region between CL and *T*. *mcilwraithi* at CY yielded no specimens of the genus. The type-locality of *T*. *mcilwraithi* is, thus, almost two degrees of latitude north of previous records for the genus. Collection records by Dyne and Wallace [79] did not extend North of Cooktown. Biological diversity of the Cape York Peninsula is understudied and our results suggest that biologists interested in understanding phylogenetic, biogeographic, and evolutionary patterns of taxa of the AWT should include taxa from this region when they are known.

There is mounting evidence for a pre-Pleistocene climatic effect on flora and fauna across the Black Mountain Corridor of the Australian Wet Tropics but we are still in the early stages of understanding the factors that may have shaped this pattern. Aridification of the Australian continent undoubtedly led to contractions of the forest and may have driven species into relictual habitats thus resulting in contracted ranges and/or massive extinctions. Diversification in isolation would have been possible during these periods of forest contractions and our data are suggestive of this. Studies of species across many taxonomic scales in this region suggests that vicariance events may be geographically, but not necessarily temporally congruent, which poses the question how common is this in other geographic regions?

Our data show that for the endemic *Terrisswalkerius* earthworms, the rainforests of the AWT have acted not only as a museum to preserve biological diversity, but also have promoted continued and rapid diversification of the genus. Interestingly we see deep divergences among species of the endemic *Terrisswalkerius* earthworms, with many lineages restricted to distinct biogeographic regions (Fig 1), but we also recover paraphyletic species suggesting recent separation without sufficient evolutionary time for ancestral polymorphisms to sort. Hybridization cannot be ruled out for the two pairs of paraphyletic species [*T*. *canaliculatus* and *T*. *terrareginae*: overlap in their distributional ranges in the Atherton Uplands (AU) and Bellenden-Ker/Bartle-Frere (BK)*; T*. *nashi* and *T*. *liber* overlap in their distributional range in the Mount Finnigan Uplands (FU)]. However, it is more probable that *T*. *liber* has evolved within *T*. *nashi* by reduction of the distance between the male pores and that reproductive isolation is not complete. In other lineages with overlapping distributions we see clear genetic differentiation such as in the Carbine Uplands (CU) where eight species are found co-occurring and the Atherton Uplands (AU) with twelve species. The results presented here show that for the *Terrisswalkerius* earthworms, the AWT are the center for ancient and ongoing diversification.

Although many vertebrate and invertebrate taxa have been examined from the Australian Wet Tropics UNESCO World Heritage Site, the *Terrisswalkerius* earthworms are the most diverse radiation that has been examined to date and the results presented here highlight the need for continued investigations across diverse groups of species and with varying depths of evolutionary history in the AWT, but also in other geographic arenas. Generally, vertebrates requiring large areas may have been subject to extinction, but when smaller vertebrates (*i*. *e*. frogs: [40] [80] [81] and invertebrate radiations have been examined [25] [28] [33] [54] [78], these data provide a richer picture of the evolutionary history of this region and suggest that these taxa had limited dispersal abilities and/or were buffered in smaller areas. The Australian Wet Tropics, which are biologically and topologically complex [82], provide an exceptional opportunity to study not only short-term evolutionary processes resulting from the Last Glacial Maximum (LGM), but also long-term processes, which affected the diversification and redistribution of biodiversity on a larger timescale. This is not unique to the AWT, with parallel questions being asked in other biologically diverse and geographically old regions such as the African tropics [83], Neotropics [84], and Madagascar [85]. Our results highlight the need for continuing evolutionary studies in this and other geographically old regions to include data from multiple levels, from individuals to higher taxonomic scales, across diverse groups of organisms thus allowing independent replicates of evolution in order to more fully unravel the complex history of the planet’s biological diversity.

## Supporting Information

S1 Fig BEAST relaxed-clock maximum clade credibility phylogeny, showing BEAST posterior probabilities followed by RAxML bootstrap supports (PP/BS as percent; some terminal nodes show only PP to avoid clutter) based on the combined data matrix with all outgroups. *Terrisswalkerius* taxa north of the Black Mountain Corridor (BMC) in grey. Node circles indicate biogeographic analyses: grey and black filled circles indicate inferred ‘vicariance’ by respectively either or both DEC and DIVA methods. Divergence scale in mya. See Jamieson *et al*. [44] and Buckley *et al*. [49] for more details on higher classification. Additional biogeographic calibration constraint nodes Megascolecidae (E) and Acanthodrilinae (B) indicated.
(TIF)

S2 Fig Phylogeny and branches labeled as implemented in DEC and DIVA biogeographic analyses. Branch labels correspond to values in S3 Table.
(TIF)

S3 Fig BEAST v1.4.8 analysis of COII data. Maximum clade credibility chronogram from 10 million steps 10% burn-in, showing median age and 95% CI age bars (in millions of years). Labeling of groups follows Fig 2 in main text.
(TIF)

S4 Fig BEAST v1.4.8 analysis of 28S rRNA data. Maximum clade credibility chronogram from 10 million steps 10% burnin, showing 95% CI age bars (in millions of years). Labeling of groups follows Fig 2 in main text.
(TIF)

S1 File Supporting Information. Additional Materials and Methods.
(DOC)

S1 Table List of all specimens, collection accession numbers and GenBank accession numbers. “X” denotes missing sequence information for taxon.
(DOC)

S2 Table Biogeographic analysis constraint matrix for DEC and DIVA. (DOC)

S3 Table Summary of DEC and DIVA biogeographic analyses following labeling on S2 Fig. (DOC)
