# Supplementary figures and images for: An Ancient Divide in a Contiguous Rainforest: Endemic Earthworms in the Australian Wet Tropics
Source: PLoS One. 2015 Sep 14;10(9):e0136943. doi: 10.1371/journal.pone.0136943 (PMC4569478; doi:10.1371/journal.pone.0136943)

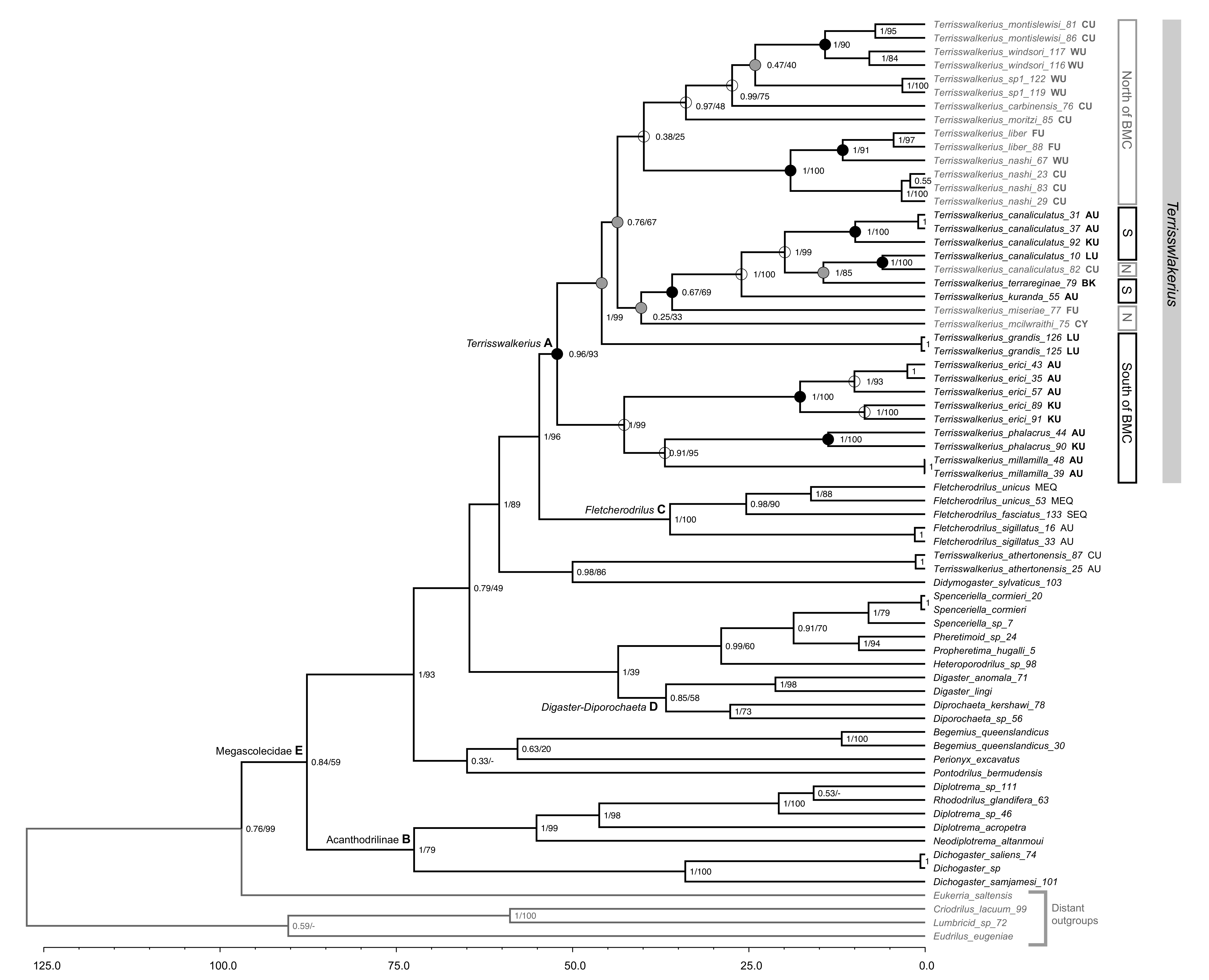

Supplement: S1 Fig — Terrisswalkerius taxa north of the Black Mountain Corridor (BMC) in grey. Node circles indicate biogeographic analyses: grey and black filled circles indicate inferred ‘vicariance’ by respectively either or both DEC and DIVA methods. Divergence scale in mya. See Jamieson et al. [44] and Buckley et al. [49] for more details on higher classification. Additional biogeographic calibration constraint nodes Megascolecidae (E) and Acanthodrilinae (B) indicated. (TIF) [file pone.0136943.s001.tif]

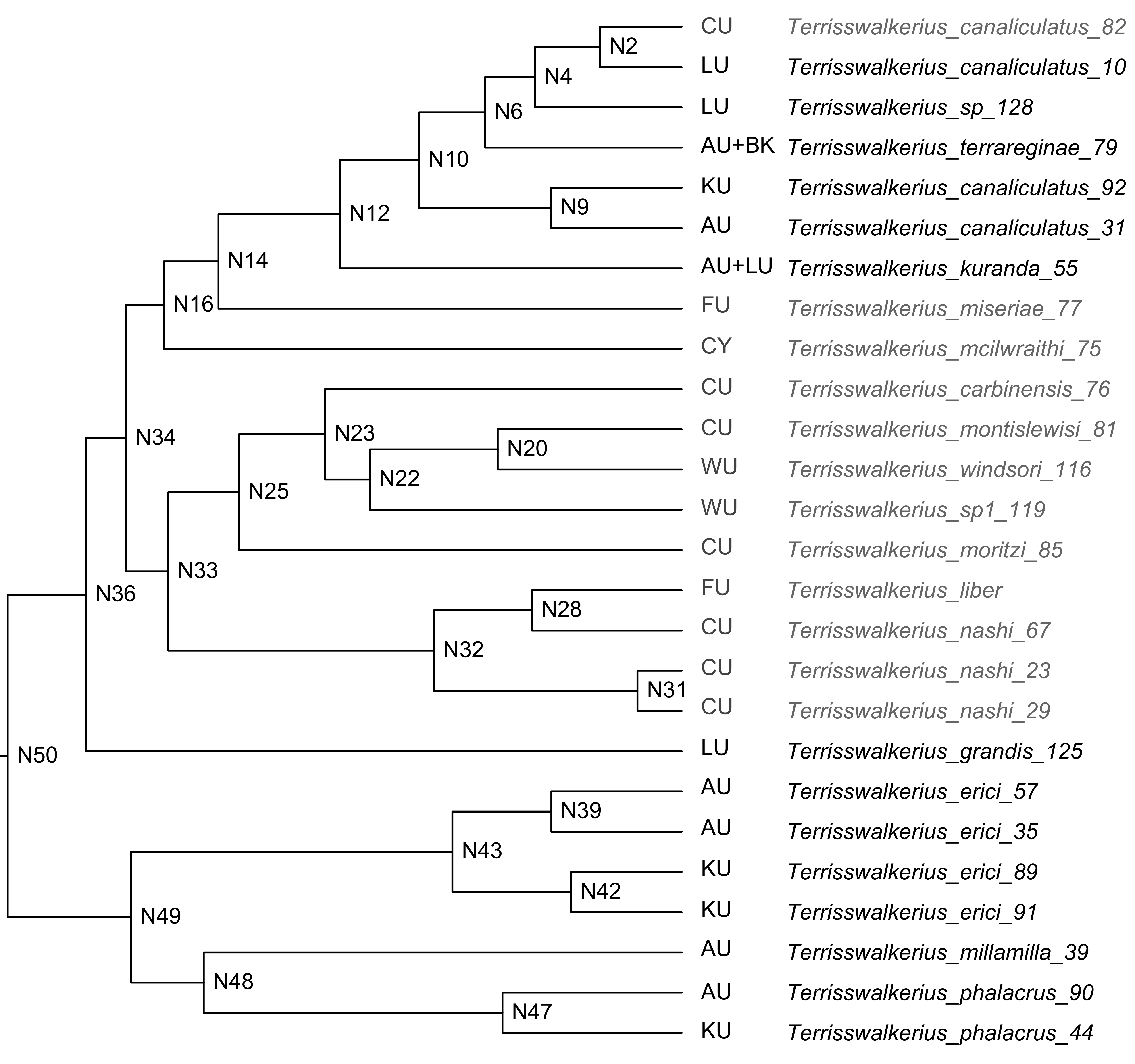

Supplement: S2 Fig — Branch labels correspond to values in S3 Table. (TIF) [file pone.0136943.s002.tif]

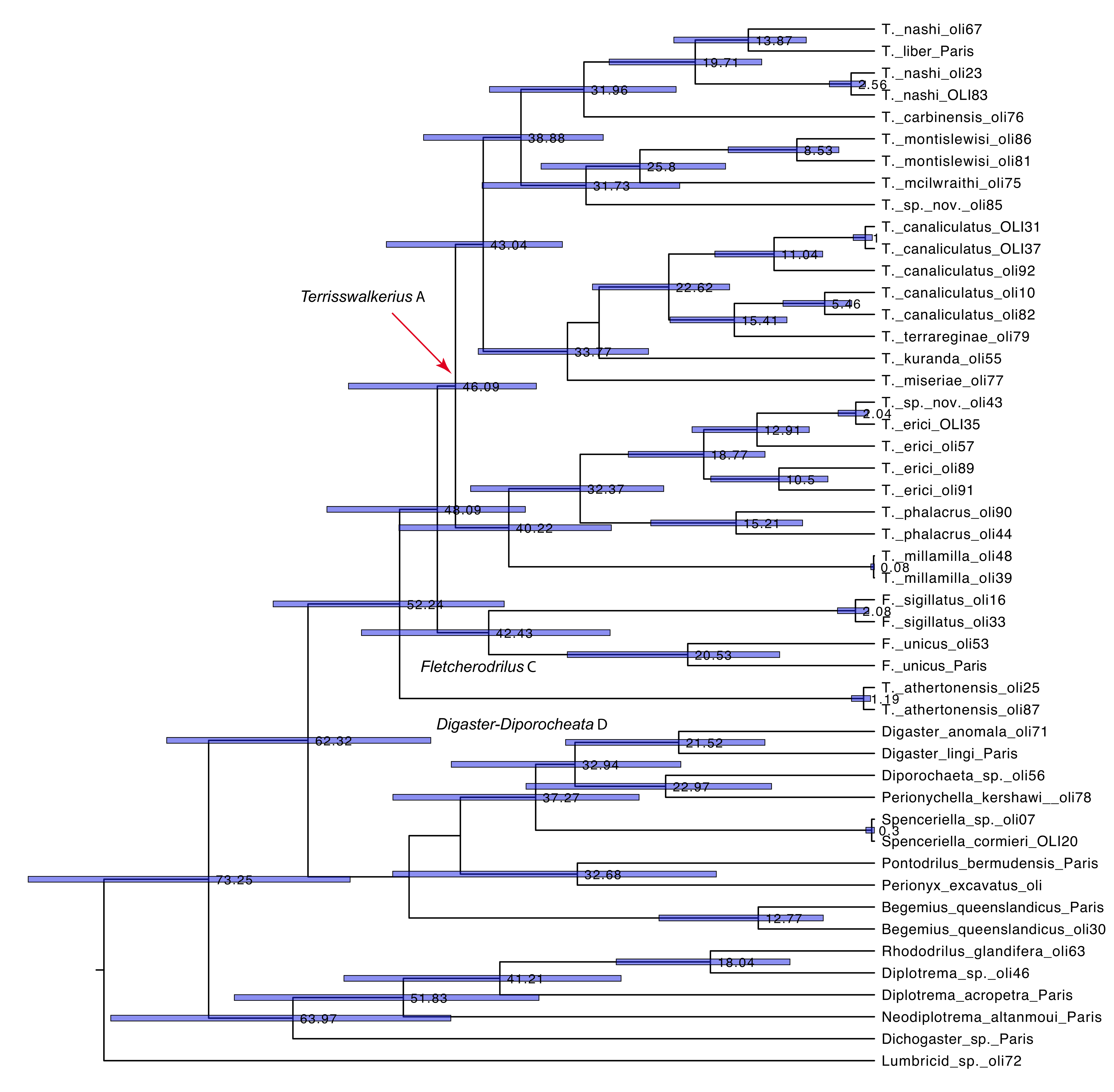

Supplement: S3 Fig — Maximum clade credibility chronogram from 10 million steps 10% burn-in, showing median age and 95% CI age bars (in millions of years). Labeling of groups follows Fig 2 in main text. (TIF) [file pone.0136943.s003.tif]

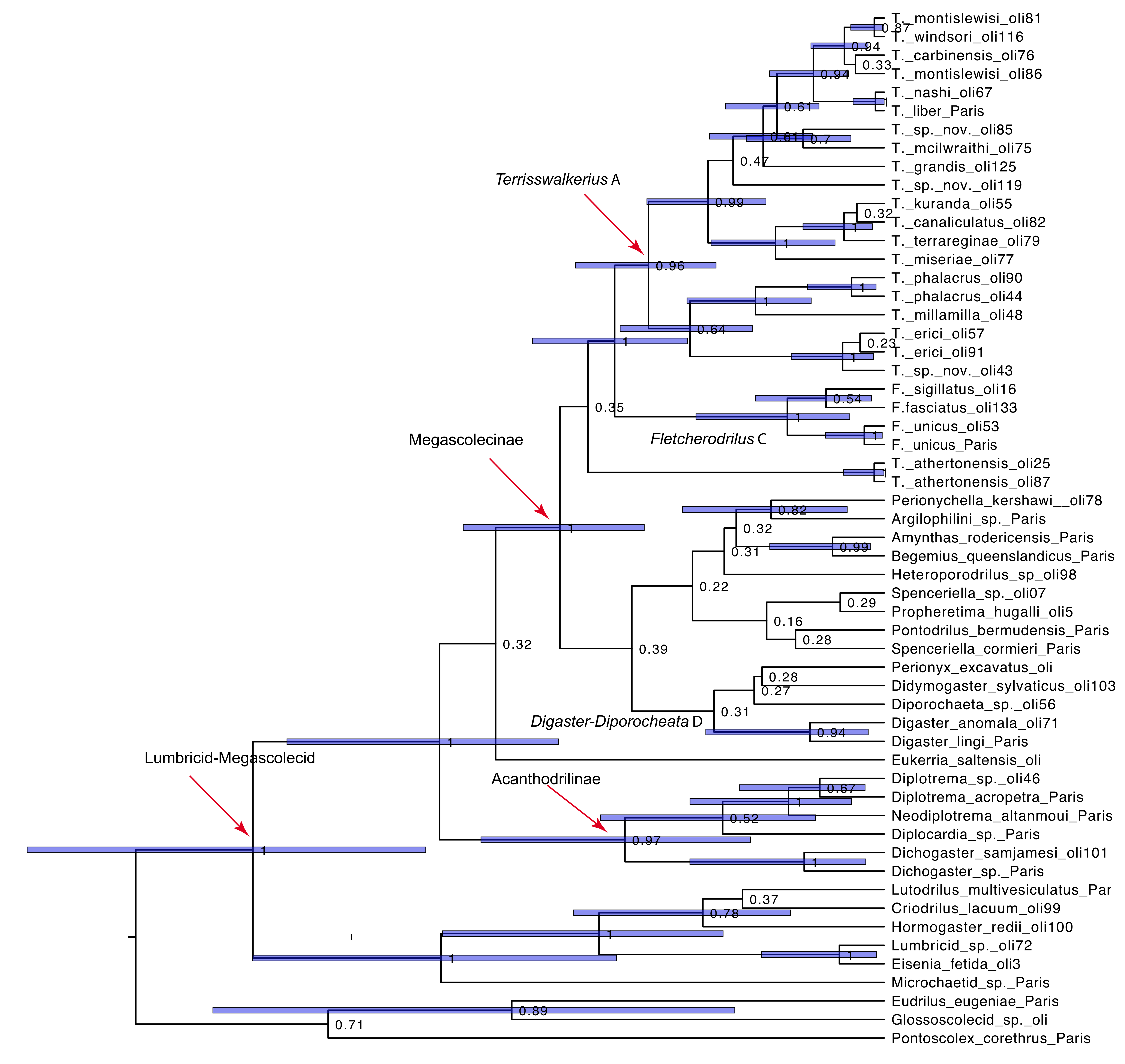

Supplement: S4 Fig — Maximum clade credibility chronogram from 10 million steps 10% burnin, showing 95% CI age bars (in millions of years). Labeling of groups follows Fig 2 in main text. (TIF) [file pone.0136943.s004.tif]
