# Supplementary material for: An Ancient Divide in a Contiguous Rainforest: Endemic Earthworms in the Australian Wet Tropics
Source: PLoS One. 2015 Sep 14;10(9):e0136943. doi: 10.1371/journal.pone.0136943 (PMC4569478; doi:10.1371/journal.pone.0136943)
Supplement: S1 File — Additional Materials and Methods. (DOC) [file pone.0136943.s005.doc]

**SUPPORTING INFORMATION - SI**

ADDITIONAL MATERIALS AND METHODS

*Polymerase Chain Reaction (PCR) Amplification.* For most specimens, four fragments were amplified via PCR (Mullis *et al*., 1987; Saiki *et al*., 1988) using specific primers for each gene region: a fragment approximately 315 base pairs (bp) in length of the small subunit (12S rRNA) ribosomal mitochondrial molecular marker; a fragment approximately 560 bp in length containing the *Cytochrome Oxidase* II (*COII*) protein encoding mitochondrial molecular marker; a fragment approximately 440 bp in length of the large subunit (16S rRNA) ribosomal mitochondrial molecular marker; a fragment approximately 740 bp in length of the large subunit (28S rRNA) ribosomal nuclear molecular marker. Nucleotide sequences were first aligned using CLUSTAL W (Thompson *et al*. 1994). These were then modified according to accepted secondary structure models for 12S rRNA (Hickson *et al*. 1996) and 16S rRNA (see De Rijk *et al*. 1999). Several regions of ambiguous alignment were then excluded from subsequent analyses. Within 28S rRNA three regions were removed: one in the D1 domain (10 bp between the B13 and B13 1 stems) and two in the D2 domain (10 and 12 bp) leaving 695 sites. For 12S rRNA, four loop regions of variable length between stem regions 40’-39’, 42- 42’, 47-47’ and 48-48’ totalling 30-50 bp were removed, leaving 315 sites. Using the nomenclature of De Rijk *et al*. (1999) small sections of the E25 and G3 loops were removed from the 16S rRNA alignment, totalling 10-20 bp, leaving 435 sites.

*Phylogenetic Analysis.* Additional phylogenetic inference was conducted with PAUP*4.0b10 (Swofford, 2001), GARLI v0.951 (Zwickl, 2006), and MrBayes v3.1.1 (Huelsenbeck & Ronquist, 2001). In order to evaluate the fit of the data, likelihood analyses were conducted using the complete concatenated data set with GARLI v0.951 (Zwickl, 2006) and MrBayes v3.1.1 (Huelsenbeck & Ronquist, 2001). A series of nested hypotheses in which the null hypothesis (H_0_) is a special case of the alternative hypothesis (H_1_) were performed on various nucleotide substitution models using the likelihood ratio test (LRT) within Modeltest 3.06 (Posada & Crandall, 1998). A maximum likelihood search was implemented in GARLI v0.951 (Zwickl, 2006) with model parameters being estimated during the run, with genthreshfortopoterm = 10,000,000; scorethreshforterm = 0.05; significanttopochange = 0.05; stopgen = 10,000,000; and stoptime = 10,000,000. This process was implemented several times to insure the topology converged on the same maximum likelihood tree. A single GTR+Γ+I model of sequence evolution was assumed to underlie all gene regions. To test the robustness of the final maximum likelihood (ML) tree, a bootstrap analysis was performed in GARLI v0.951 (Zwickl, 2006) for 500 pseudoreplicates.

*Relative Dating Analyses.* In addition to the combined data analysis, relaxed-clock relative divergence dating was conducted on two additional data matrices: the nuclear 28SrDNA alone; and the mitochondrial COI alone. These were run in BEAST v1.4.8 (Drummond & Rambaut 2007) using the uncorrelated lognormal relaxed-clock model, Yule speciation (node-height) prior, GTR+Γ sequence evolution model, and running 10 million steps with a 10% burn-in. These analyses were arbitrary calibrated by a fixed rate of 1, and the posterior distribution of ages of several key splits expressed as relative to the age of the monophyletic *Terrisswalkerius* (node A). Separate COII and 28S trees are shown in Figures S2 and S3, and relative dating results are in Figure 3.

**SUPPORTING INFORMATION REFERENCES**

Buckley TR, James S, Allwood J, Bartlam S, Howitt R, Prada R (2011) Phylogenetic analysis of New Zealand earthworms (Oligochaeta: Megascolecidae) reveals ancient clades and cryptic taxonomic diversity. Mol Phylogen Evol 58: 85-96.

De Rijk P, Robbrecht E, de Hoog S, Caers A, Van de Peer Y, De Wachter R (1999) Database on the structure of large subunit ribosomal RNA. Nucleic Acids Res 27: 174-178.

Drummond AJ, Rambaut A (2007) BEAST: Bayesian evolutionary analysis by sampling trees. BMC Evol Biol 7: 214.

Hickson RE, Simon C, Cooper A, Spicer GS, Sullivan J, Penny D (1996) Conserved sequence motifs, alignment, and secondary structure for the third domain of animal 12S rRNA. Mol Biol Evol 13: 150-169.

Huelsenbeck JP, Ronquist F (2001) MRBAYES: Bayesian inference of phylogeny. Bioinformatics 17: 754-755.

Jamieson BGM, Tillier S, Tillier A, Justine J-L, Ling E, James S, McDonald K, Hugall AF (2002) Phylogeny of the Megascolecidae and Crassiclitellata (Annelida, Oligochaeta): combined versus partitioned analysis using nuclear (28S) and mitochondrial (12S, 16S) rRNA. Zoosystema 24: 707-734.

Loader SP, Pisani D, Cotton JA, Gower DJ, Day JJ, Wilkinson M (2007) Relative time scales reveal multiple origins of parallel disjunct distributions of African caecilian amphibians. Biol Lett 3: 505-508.

Mullis K, Faloona F, Scharf S, Saiki R, Horn G, Erlich HA (1987) Specific enzymatic amplification of DNA in vitro: the polymerase chain reaction. Cold Spring Harbor Symp Quant Biol 51: 263-273.

Posada D, Crandall KA (1998) Modeltest: testing the model of DNA substitution. Bioinformatics 14: 817-818.

Saiki RK, Gelfand DH, Stoffel S, Scharf SJ, Higuchi R, Horn GT, Mullis KB, Erlich HA (1988) Primer-directed enzymatic amplification of DNA with a thermostable DNA polymerase. Science 239: 487-491.

Swofford DL (2001) PAUP*Star (ver 4.0b10). Sinauer, Sunderland, MA, USA.

Thompson JD, Higgins DG, Gibson TJ (1994) CLUSTAL W: improving the sensitivity of progressive multiple sequence alignment through sequence weighting, positions-specific gap penalties and weight matrix choice. Nucleic Acids Res 22: 4673-4680.

Williams SE, Pearson RG, Walsh PJ (1996) Distributions and biodiversity of the terrestrial vertebrates of Australia’s wet tropics: a review of current knowledge. Pacific Conserv Biol 2: 327-362.

Zwickl DJ (2006) GARLI: Genetic Algorithm for Rapid Likelihood Inference, Version 0.951: http://www.bio.utexas.edu/faculty/antisense/g
