# Supplementary material for: An Ancient Divide in a Contiguous Rainforest: Endemic Earthworms in the Australian Wet Tropics
Source: PLoS One. 2015 Sep 14;10(9):e0136943. doi: 10.1371/journal.pone.0136943 (PMC4569478; doi:10.1371/journal.pone.0136943)
Supplement: S1 Table — “X” denotes missing sequence information for taxon. (DOC) [file pone.0136943.s006.doc]

**Table S1.** List of all specimens, collection accession numbers and GenBank accession numbers. “X” denotes missing sequence information for taxon.

| **Collection#:** | **Genus:** | **Species:** | **AWT subregion/ biogeographic area:** | **12S rRNA GenBank** | **16S rRNA**  **GenBank** | ***COII* GenBank** | **28S rRNA**  **GenBank** |
| --- | --- | --- | --- | --- | --- | --- | --- |
| 117 | *Terrisswalkerius* | *windsori* | WU | FJ626958 | X | X | X |
| 116 | *Terrisswalkerius* | *windsori* | WU | AY101552 | AF406587 | X | AY048494 |
| 86 | *Terrisswalkerius* | *montislewisi* | CU | AY048437 | AF406580 | AF403442 | AY048488 |
| 81 | *Terrisswalkerius* | *montislewisi* | CU | AY048436 | X | FJ626995 | AY048500 |
| 122 | *Terrisswalkerius* | sp. 1 | WU | FJ626960 | X | X | X |
| 119 | *Terrisswalkerius* | sp. 1 | WU | FJ626962 | AF406564 | X | FJ626968 |
| 76 | *Terrisswalkerius* | *carbinensis* | CU | AY048435 | AF406584 | AF403440 | AY048482 |
| 83 | *Terrisswalkerius* | *nashi* | CU | AY048443 | X | FJ626997 | X |
| 29 | *Terrisswalkerius* | *nashi* | CU | AY048442 | X | X | X |
| 23 | *Terrisswalkerius* | *nashi* | CU | AY048441 | X | FJ626971 | X |
| 67 | *Terrisswalkerius* | *nashi* | WU | AY048438 | AF406561 | AF403438 | AY048486 |
| 54 | *Terrisswalkerius* | *liber* | FU | AY048439 | X | FJ626982 | FJ626967 |
| 88 | *Terrisswalkerius* | *liber* | FU | AY048440 | X | X | X |
| 79 | *Terrisswalkerius* | *terrareginae* | BK | AY048431 | X | FJ626994 | AY048501 |
| 82 | *Terrisswalkerius* | *canaliculatus* | CU | AY048430 | AF406562 | FJ626996 | AY048485 |
| 10 | *Terrisswalkerius* | *canaliculatus* | LU | AY048429 | X | AF403436 | X |
| 92 | *Terrisswalkerius* | *canaliculatus* | KU | AY048428 | X | FJ627000 | X |
| 37 | *Terrisswalkerius* | *canaliculatus* | AU | AY048426 | X | FJ626976 | X |
| 31 | *Terrisswalkerius* | *canaliculatus* | AU | AY048427 | X | FJ626974 | X |
| 55 | *Terrisswalkerius* | *kuranda* | AU | AY048432 | AF406581 | FJ626983 | AY048478 |
| 77 | *Terrisswalkerius* | *miseriae* | FU | AY048433 | AF406559 | FJ626992 | AY048483 |
| 85 | *Terrisswalkerius* | *moritzi* | CU | AY048455 | AF406560 | AF403441 | AY048487 |
| 75 | *Terrisswalkerius* | *mcilwraithi* | CY | AY048434 | AF406563 | FJ626991 | AY048481 |
| 126 | *Terrisswalkerius* | *grandis* | LU | FJ626963 | X | X | X |
| 125 | *Terrisswalkerius* | *grandis* | LU | AY101551 | AF406566 | X | AY048495 |
| 90 | *Terrisswalkerius* | *phalacrus* | KU | AY048450 | AF406577 | AF403443 | AY048490 |
| 44 | *Terrisswalkerius* | *phalacrus* | AU | AY048449 | X | FJ626979 | AY101556 |
| 48 | *Terrisswalkerius* | *millamilla* | AU | AY048452 | AF406565 | AF403437 | AY048476 |
| 39 | *Terrisswalkerius* | *millamilla* | AU | AY048451 | X | FJ626977 | X |
| 91 | *Terrisswalkerius* | *erici* | KU | AY048448 | X | FJ626999 | AY048502 |
| 89 | *Terrisswalkerius* | *erici* | KU | AY048447 | X | FJ626998 | X |
| 57 | *Terrisswalkerius* | *erici* | AU | AY048446 | AF406576 | AF403434 | AY048489 |
| 35 | *Terrisswalkerius* | *erici* | AU | AY048445 | X | FJ626975 | X |
| 43 | *Terrisswalkerius* | *erici* | AU | AYO48444 | X | FJ626978 | FJ626966 |
| 133 | *Fletcherodrilus* | *fasciatus* | SEQ | AY101548 | X | X | AY048503 |
| 26 | *Fletcherodrilus* | *unicus* | MEQ | AY048423 | AF406558 | AF403433 | AY101565 |
| 53 | *Fletcherodrilus* | *unicus* | SEQ | AY101547 | X | FJ626981 | AY048474 |
| 33 | *Fletcherodrilus* | *sigillatus* | AU | AY048425 | X | AF403432 | X |
| 16 | *Fletcherodrilus* | *sigillatus* | AU | AY048424 | AF406588 | FJ626970 | AY048473 |
| 87 | *Terrisswalkerius* | *athertonensis* | CU | AY048454 | X | AF403435 | AY048504 |
| 25 | *Terrisswalkerius* | *athertonensis* | AU | AY048453 | AF406585 | FJ626972 | FJ626965 |
| 103 | *Didymogaster* | *sylvaticus* | NSW | AY101554 | AF406575 | X | AY048491 |
| 7 | *Spenceriella* | sp. | NSW | AY048457 | AF406572 | AF403431 | AY048475 |
| 21 | *Spenceriella* | *cormieri* | BR | AY048458 | AF406589 | X | AY101564 |
| 20 | *Spenceriella* | *cormieri* | BR | AY048457 | X | AF403431 | X |
| 63 | *Rhododrilus* | *glandifera** | AU | AY048467 | X | FJ626987 | X |
| 5 | *Propheretima* | *hugalli* | NSW | AY101550 | X | X | AY048505 |
| 62 | *Pontodrilus* | *litoralis* | Western AUST | AY048463 | AF406586 | FJ626986 | AY101576 |
| 24 | *Pheretimoid* | sp. | BR | FJ626961 | X | X | X |
|  | *Perionyx* | *excavatus* | cultured (India) | AY048456 | AF406582 | FJ627002 | AY048499 |
| 78 | *Diprochaeta (=Perionychella)* | *kershawi* | TAS | AY048461 | AF406567 | FJ626993 | AY048484 |
| 69 | *Neodiplotrema* | *altanmoui* | FNQ | AY048468 | AF406569 | FJ626989 | AY101574 |
| 72 | *Lumbricid* | sp. | SEQ | AY048472 | U24570 (mt genome) | U24570 | AY048498 |
| 98 | *Heteroporodrilus* | sp. | SEQ | AY101553 | AF406579 | X | AY048497 |
|  | *Eukerria* | *saltensis* | cultured (South America) | AY101546 | AF406590 | X | AY048496 |
|  | *Eudrilus* | *eugeniae* | cultured (Africa) | AY048471 | X | X | AY101568 |
| 56 | *Diporochaeta* | sp. | TAS | AY048460 | AF406574 | FJ626984 | AY048479 |
| 46 | *Diplotrema* | sp. | AU | AY048466 | AF406570 | FJ626980 | AY048477 |
| 111 | *Diplotrema* | sp. | FNQ | FJ626959 | X | X | X |
| 93 | *Diplotrema* | *acropetra* | FNQ | AY048469 | AF406568 | FJ627001 | AY101573 |
| 68 | *Digaster* | *lingi* | BR | AY048459 | X | FJ626988 | AY101561 |
| 71 | *Digaster* | *anomala* | SEQ | AY048462 | AF406583 | AF403439 | AY048480 |
| 73 | *Dichogaster* | sp. | cultured | AY048470 | AF406573 | FJ626990 | AY48493 |
| 74 | *Dichogaster* | *saliens* | cultured (Africa) | FJ626964 | X | X | X |
| 101 | *Dichogaster* | *m.s.sp. samjamesi* | Carribean | AY101549 | AF406571 | X | AY101555 |
| 99 | *Criodrilus* | *lacuum* | Algeria | AY101545 | X | X | AY048492 |
| 59 | *Begemius* | *queenslandicus* | AU | AY048464 | X | FJ626985 | X |
| 30 | *Begemius* | *queenslandicus* | CU | AY048465 | AF406578 | FJ626973 | AY101563 |

* Now *Diplotrema glandifera*; Dyne and Jamieson (2004)
