# Supplementary material for: An Ancient Divide in a Contiguous Rainforest: Endemic Earthworms in the Australian Wet Tropics
Source: PLoS One. 2015 Sep 14;10(9):e0136943. doi: 10.1371/journal.pone.0136943 (PMC4569478; doi:10.1371/journal.pone.0136943)
Supplement: S2 Table — (DOC) [file pone.0136943.s007.doc]

**Table S2.** Dispersal constraint matrix implemented in the biogeographic analysis within the software packages LAGRANGE (Ree et al. 2005; Ree & Smith 2008) and DIVA (Yu et al. 2010).

|  | AU | BK | CU | CY | FU | KU | LU | WU |
| --- | --- | --- | --- | --- | --- | --- | --- | --- |
| BK | 1 | - |  |  |  |  |  |  |
| CU | 0 | 0 | - |  |  |  |  |  |
| CY | 0 | 0 | 1 | - |  |  |  |  |
| FU | 0 | 0 | 1 | 1 | - |  |  |  |
| KU | 1 | 1 | 0 | 0 | 0 | - |  |  |
| LU | 1 | 1 | 1 | 0 | 0 | 1 | - |  |
| WU | 0 | 0 | 1 | 1 | 1 | 0 | 0 | - |
