# Supplementary material for: An Ancient Divide in a Contiguous Rainforest: Endemic Earthworms in the Australian Wet Tropics
Source: PLoS One. 2015 Sep 14;10(9):e0136943. doi: 10.1371/journal.pone.0136943 (PMC4569478; doi:10.1371/journal.pone.0136943)

**Table S3.** Biogeographic inferences from the DEC and DIVA analyses for nodes in Figure S2. DEC p-vicar is the marginal likelihood for all between exclusive regions solutions.


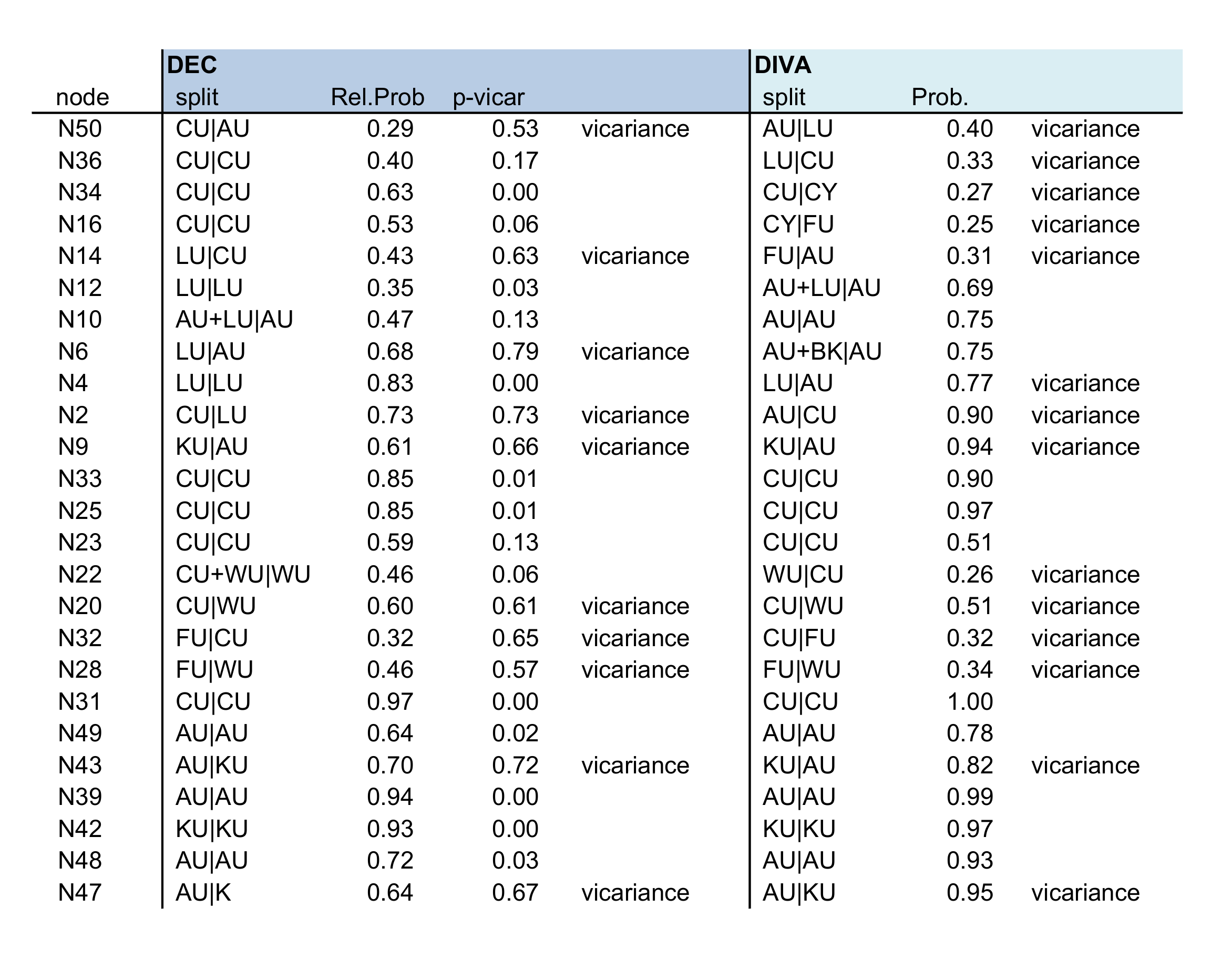

Supplement: S3 Table — (DOC) [file pone.0136943.s008.doc]
